# Case report: JAK1/2 inhibition with baricitinib in the treatment of STING-associated vasculopathy with onset in infancy

**DOI:** 10.1186/s12969-023-00916-6

**Published:** 2023-10-26

**Authors:** Jianqiang Wu, Qing Zhou, Hua Zhou, Meiping Lu

**Affiliations:** 1 Department of Rheumatology Immunology and Allergy, Children’s Hospital, School of Medicine, Zhejiang University, National Clinical Research Center for Child Health, 3333, Binsheng Road, Hangzhou, 310052 China; 2 Life Sciences Institute, Zhejiang University, Hangzhou, China; 3 Department of Respiratory and Critical Care Medicine, The First Affiliated Hospital, Zhejiang University School of Medicine, Hangzhou, China

**Keywords:** STING-associated vasculopathy with onset in infancy (SAVI), Type I interferonopathy, Janus kinase inhibitors (JAK inhibitors), Interstitial lung Disease (ILD)

## Abstract

**Background:**

Gain-of-function mutations in *STING1* (also known as TMEM173) which result in constitutive activation of STING, have been reported to cause STING-associated vasculopathy with onset in infancy (SAVI). Although a wider spectrum of associated manifestations and perturbations in disease onset have been observed since its description, the genotype-phenotype correlations are not definite, and there is no established treatment protocol for SAVI.

**Case presentation:**

Herein, we report a kindred, heterozygous STING mutation (p.V155M) in which the 2-year-old proband suffered from severe interstitial lung disease (ILD) while her father was initially misdiagnosed with connective tissue disease associated with ILD at an adult age. Baricitinib was initiated after the diagnosis of SAVI in the proband combined with steroids, and during the 14-month follow-up, the respiratory symptoms were improved. However, as the improvement of laboratory indicators was limited, especially in autoimmune indices, and the lung CT images remained unaltered, it seems that JAK1/2 inhibition was unsatisfactory in completely controlling the inflammation of the disease in our study.

**Conclusions:**

Baricitinib was shown to elicit some effect on the ILD but failed to control the inflammation of the disease completely. Further exploration of JAK inhibitors or other therapeutic strategies are needed to more optimally treat this inflammatory disease.

**Supplementary Information:**

The online version contains supplementary material available at 10.1186/s12969-023-00916-6.

## Background

Gain-of-function mutations in *STING1* (human transmembrane protein 173 gene), which result in constitutive activation of STING, have been reported to cause an autoinflammatory syndrome termed SAVI (STING-associated vasculopathy with onset in infancy) [1]. SAVI is an interferonopathy that typically manifests with severe cutaneous involvement, progressive pulmonary lesions mainly associated with interstitial lung disease (ILD), and systemic inflammation due to type I interferon (IFN) overproduction [1, 2]. However, the therapeutic management of SAVI is challenging. There is presently no established treatment protocol for SAVI. Conventional immunosuppressive therapies failed to treat most patients [1, 2]. Based on its pathogenesis, the use of Janus kinase (JAK) inhibitors has recently been presumed to be a promising therapy for SAVI [3]. We herein reported a case of SAVI with severe pulmonary involvement, in which treatment with the JAK1/2 inhibitor baricitinib achieved some expected responses, and then summarized the characteristics of disease presentations for early diagnosis and prognosis.

## Case presentation

**Fig. 1:**
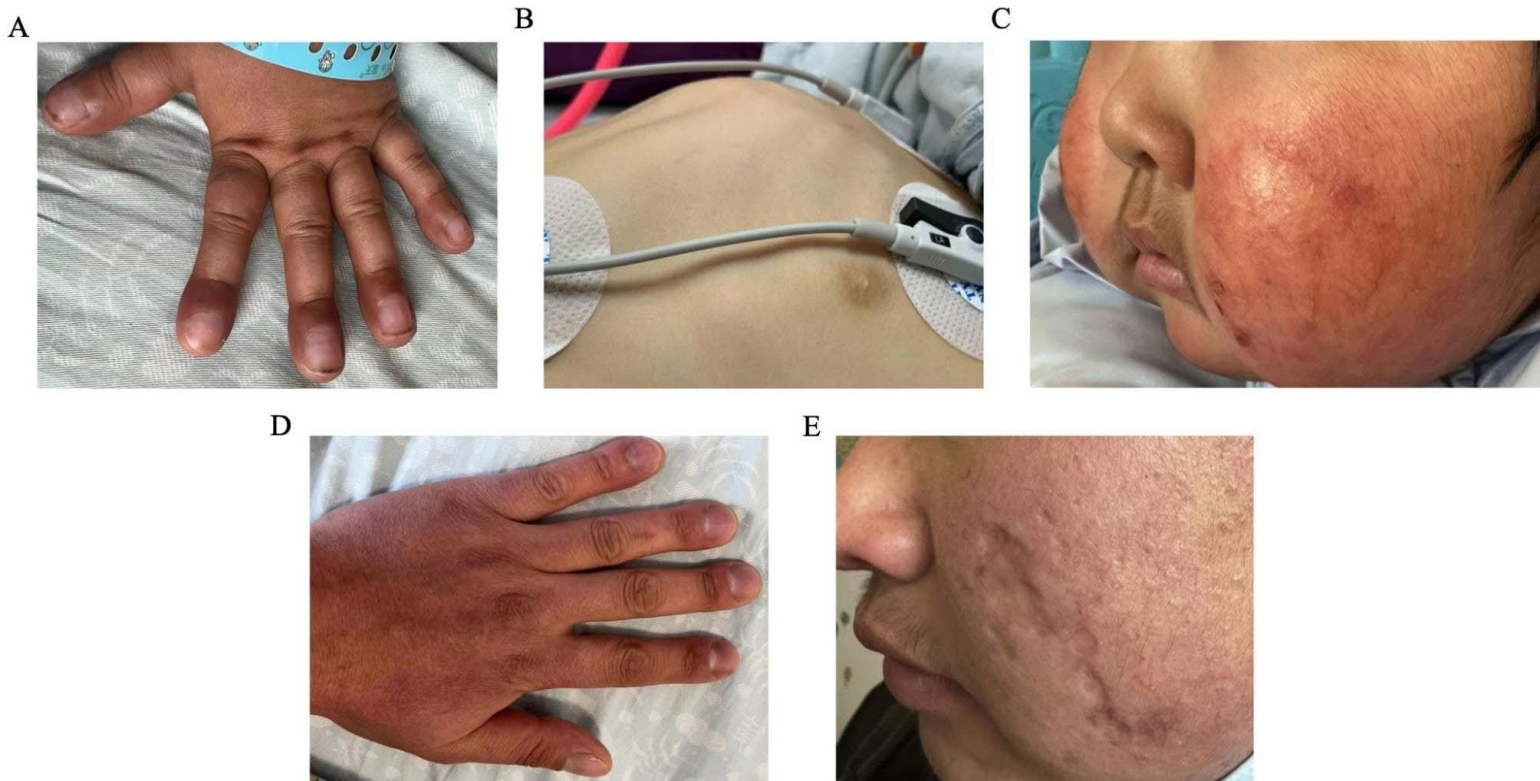
Clinical manifestations of the proband and her father. (A) Clubbing fingers of the proband. (B) Pigeon breast deformity of the proband. (C) Facial rashes of the proband. (D, E) The presence of clubbing fingers and facial tissue loss in the proband’s father

**Fig. 2:**
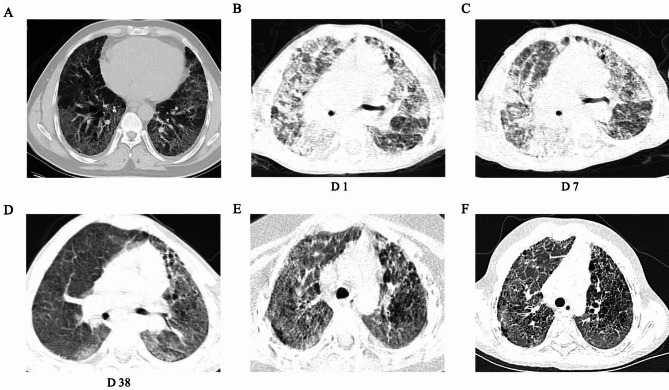
Chest computed tomography findings. (A) CT scan of the proband’s father shows interstitial pulmonary inflammation. (B-F) CT scan of the proband (B) before treatment (Day 1); (C) after treatment of antibiotics and IVIG (Day 7); (D) after treatment of MP pulse, and 1 week after baricitinib (Day 38) (E) after severe pneumonia in the 4th month; (F) 14 months after first discharge

A 2-year-6-month-old girl was referred to our hospital due to complaints of fever, cough, and tachypnea 10 days prior to her presentation. Upon admission, physical examinations revealed a body temperature of 38.5 °C, and percutaneous oxygen saturation (SpO2) was 67% in room air. Chest auscultation revealed fine crackles in the posterior lung fields. Clubbing fingers, pigeon breast deformity, and chilblain-like rash on her face were observed (Fig. 1A, B, C). Her anterior fontanelle was not closed (0.3 cm×0.3 cm). Other cutaneous manifestations were not observed. The patient displayed a height of 87 cm (-1.08 SD) and a weight of 11.5 kg (-0.82 SD). The patient experienced recurrent respiratory tract infections since the age of 6 months (once a month), and fatigue after activities was noticed beginning at 2 years of age. Family history found that her 27-year-old father had chest tightness and shortness of breath after activity beginning at 22 years of age. The clubbed fingers presented in all limbs, and mild tissue loss showed up on his face (Fig. 1D, E). Chest high-resolution computed tomography (HRCT) showed interstitial pneumonia with pulmonary cyst (Fig. 2A), and pulmonary function tests indicated mixed ventilation dysfunction. Combined with positive levels of antinuclear antibodies (ANA) and signal recognition particles (SRP) autoantibody, he was initially diagnosed with connective tissue disease (CTD) associated with ILD. After receiving combined therapy with cyclophosphamide and hydroxychloroquine for half a year, the symptoms have been improved while continuing to take hydroxychloroquine. However, about three years ago, the drug was stopped for unknown reasons, and the symptoms of dyspnea reappeared and slowly progressed.

Laboratory examinations of the female patient found an elevation in the levels of C-reactive protein (CRP, 54.19 mg/L, reference range: <8 mg/L), erythrocyte sedimentation rate (ESR, 89 mm/H, reference range: <20 mm/H), and white blood cell count (WBC, 16.3 × 10^3^/mm^3^, reference range: 4 to 10 × 10^3^/mm^3^). There was no elevation of hepatobiliary enzymes, and her renal and thyroid functions were normal. The autoimmune workup revealed elevated levels of IgG (25.3 g/L), IgA (3.26 g/L), and IgM (1.69 g/L) despite normal levels of C3 and C4 complement. Decreased CD4 + T lymphocyte counts and normal CD8 + T lymphocyte counts were identified. This patient was positive for auto-antibodies, including high-titer ANA (1:1000), anti-neutrophil cytoplasmic antibodies (ANCA), rheumatoid factor (RF), and anti-cyclic citrullinated peptide (CCP) antibodies. Cytokine testing showed an elevated interleukin (IL)-6 level (62.4 pg/ml, reference range: 1.7 to 16.6 pg/ml). Pathology tests were negative, and malignancy was ruled out. HRCT scanning of the chest exhibited diffuse ground-glass opacities (Fig. 2B). Brain magnetic resonance imaging showed widened cerebral sulci and enlarged extra-axial spaces. No abnormalities were found in the electrocardiogram or the echocardiogram. The results of abdominal ultrasound and vascular ultrasonography were normal.

**Fig. 3:**
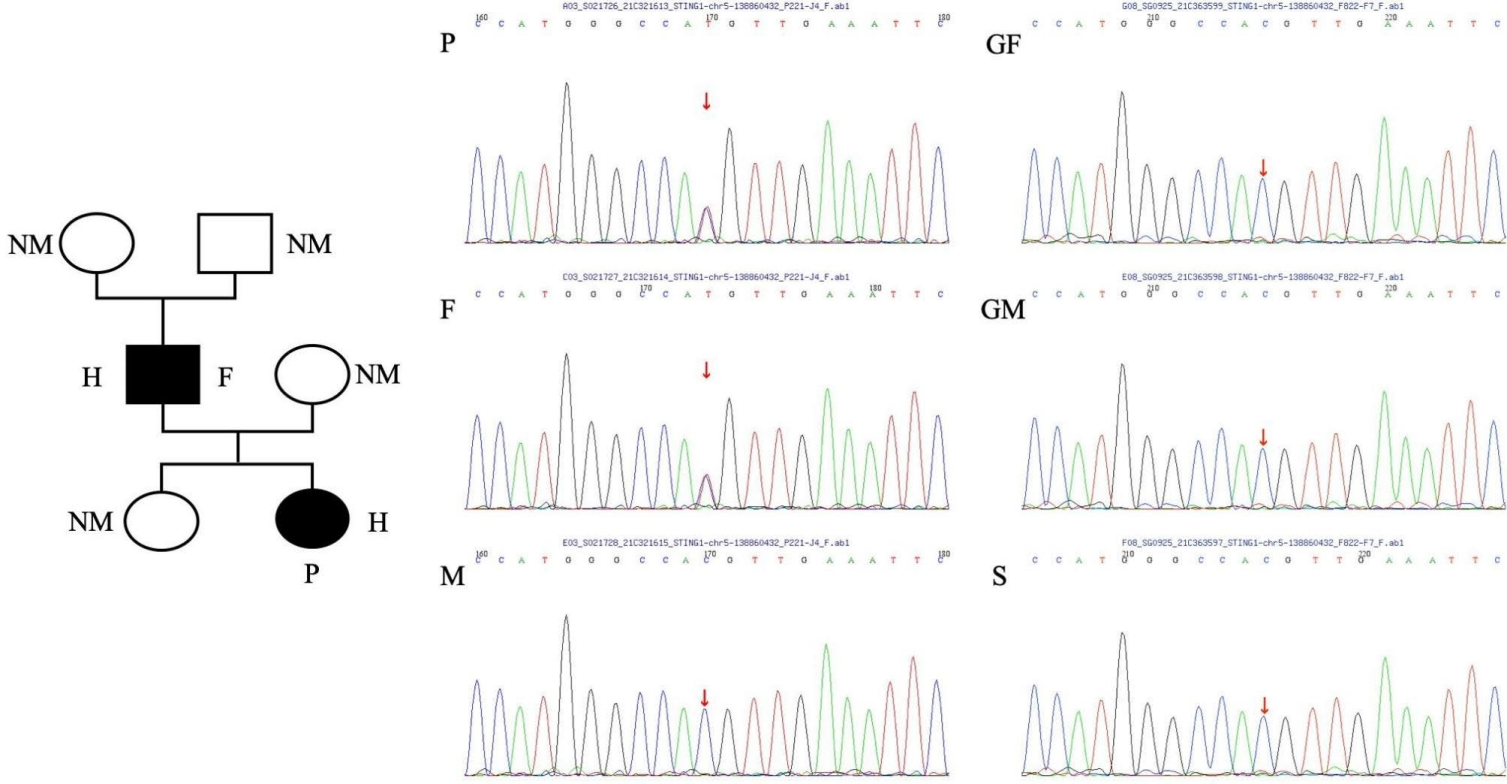
Pedigree of the patient with mutations in TMEM173. (A) Solid symbols indicate affected relatives, open symbols indicate unaffected relatives, squares represent female persons, and circle represent male persons. H denotes heterozygous mutated gene, NM denotes nonmutated gene. P denotes the proband, F denotes the father of the proband. (B) Whole-exome sequencing (WES) filtering was performed on patients and his relatives. M denotes the mother of the proband, S denotes the sister of the proband, GF denotes the grandfather of the proband, GM denotes the grandmother of the proband

**Fig. 4:**
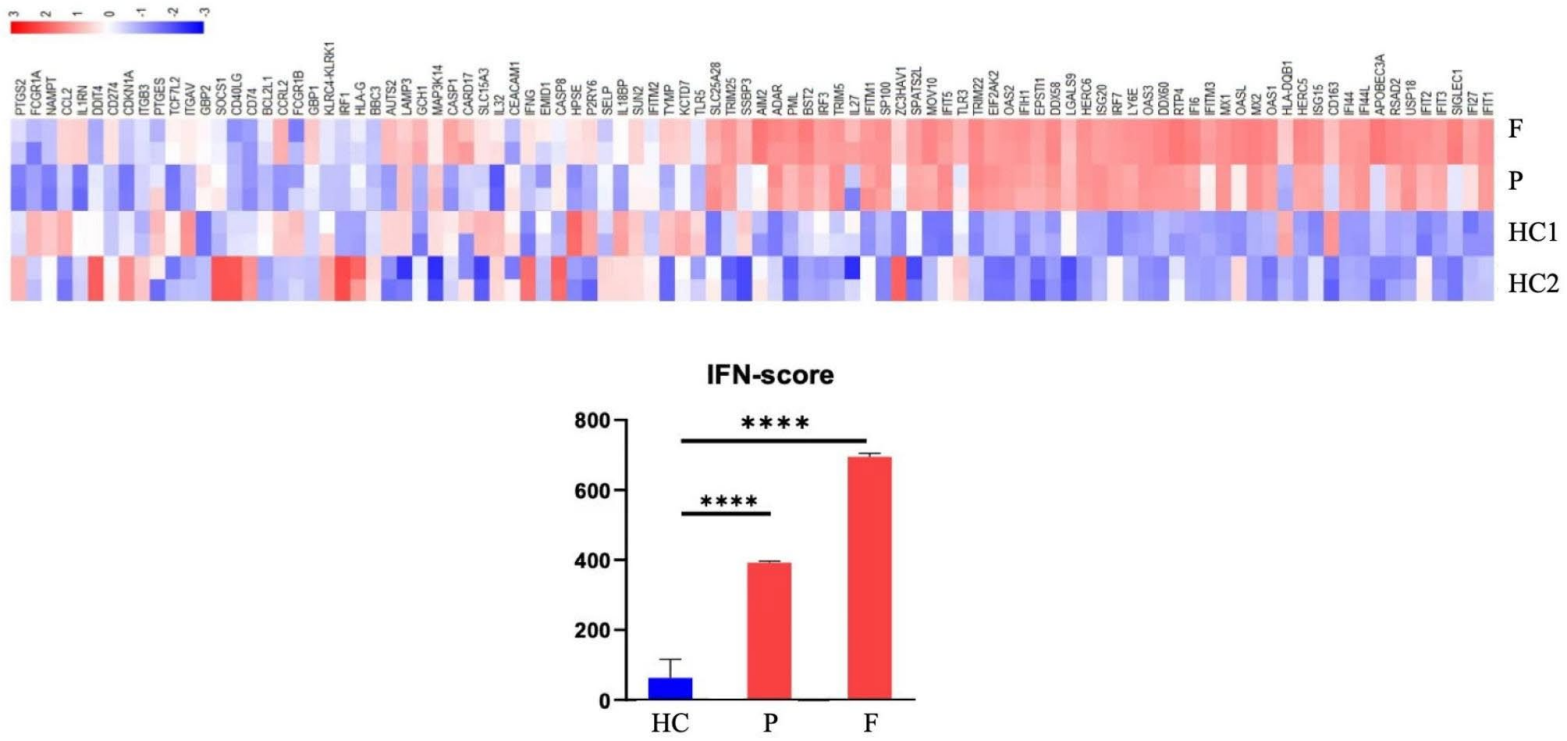
IFN score from whole blood for the proband before receiving baricitinib treatment, her father under the treatment of tofacitinib, and healthy controls. P denotes the proband, F denotes the father of the proband, HC denotes a healthy control

The female (child) patient was initially diagnosed with ILD accompanied by severe pneumonia and received anti-infection treatment with intravenous immunoglobulin (IVIG) (2 g/kg). However, the patient’s symptoms did not respond to the treatment (Fig. 2C), and she experienced hypoxic respiratory failure on Day 12, which required endotracheal intubation and mechanical ventilation. Therapies with methylprednisolone (MP) pulse (30 mg/kg/d×5d) were started on Day 13 followed by intravenous MP in 4 mg/kg daily dosage combined with a second course of IVIG (1 g/kg/d×3d). After the treatment, symptoms of dyspnea improved, and the ventilator was withdrawn on Day 22. At the same time, whole-exome sequencing was also performed, and genetic analysis by Sanger sequencing confirmed a heterozygous mutation (c.463G > A p.V155M) in Exon 5 of STING1 (NM_198282), which had been described to cause SAVI. The mutation was inherited from her father (Fig. 3). RNA sequencing on the proband and her father was conducted, which found that the expression of genes related to the interferon pathway was significantly upregulated compared to that of healthy controls (Fig. 4). After the diagnosis of SAVI, baricitinib (2.5 mg/day gradually increased to 3 times a day within 2 weeks) was added with prophylactic trimethoprim/ sulfamethoxazole, and the dose of MP was reduced to 2 mg/kg/d on Day 32. Under appropriate treatment, the patient’s symptoms of fever, tachypnea, breathlessness, and cyanosis improved, while the repeated chest HRCT showed a better result (Fig. 2D). Low-flow oxygen support (SpO2 at 94–95% in room air) was required, and the patient was discharged on Day 48, following the treatment of MP (1 mg/kg/d) and baricitinib (reduced to 2 mg three times a day). The female (child) patient was followed for 14 months after discharge. She was infected with severe pneumonia in the fourth month. Although the respiratory symptoms improved after antibiotic therapy, combined treatment with pirfenidone (2.5 mg twice-daily dosage) was administered due to the progressed interstitial pneumonia observed in HRCT (Fig. 2E). In the sixth month, we reduced the MP dose to 2 mg/d (0.13 mg/kg/d), which has been maintained until now. During the follow-up, the lung HRCT scan was stable after treatment (Fig. 2F); however, there was no obvious improvement. Laboratory indicators are still positive for ANA (1:1000), p-ANCA, RF, and CCP, while high IgG, IgA, and IgM immunoglobulin counts and low CD4 + T lymphocyte counts remain. Levels of inflammatory markers like CRP and IL-6 were normal, while levels of ESR (29 mm/H) and IFN-γ (20.1 pg/ml) were slightly elevated. Nevertheless, the evaluation of clinical features showed that the respiratory signs and symptoms have almost disappeared, except for slight dyspnea after strenuous exercise. The patient’s height increased from 87 cm (-1.08 SD) to 100 cm (-0.66 SD), weight increased from 11.5 kg (-0.82 SD) to 15.5 kg (-0.28 SD), and the fontanel closed. The little girl now lives a normal life.

After diagnosis, the male (father) patient received oral steroids and tofacitinib combination therapy for over a year, but there was no significant improvement in pulmonary symptoms. Six months ago, tofacitinib was changed to baricitinib combined with oral steroids, and the patient felt an improvement in breathing difficulties. However, similarly, there was no improvement in pulmonary imaging findings.

## Discussion and conclusions

In 2014, a new Mendelian disease, SAVI, was classified as a novel type I interferonopathy, in which enhanced type I IFN (IFN-I) pathway activation plays a key role [1]. The IFN-I pathway produces innate immunity and antiviral proteins by sensing aberrant dsDNA via cGAS-cGAMP-STING axis, which activates IRF3 and IFN-I expression [4–6]. In addition, cGAS–STING also mediates NF-κB activation and interacts with various cell death and senescence pathways [4, 5].

Herein, we described a case of SAVI caused by the p.V155M heterozygous mutation of TMEM173, which was inherited from the female proband’s father. To our knowledge, more than 70 SAVI cases with autosomal-dominant SAVI have been published since 2014 [6]. Disease onset of SAVI usually manifests by the age of 1 year [7], ranging from the neonatal period to adulthood, though adult onset is rarely observed. Vasculopathy resulting from vasculitis and endothelial cell death is a hallmark of SAVI. The major clinical characteristics of SAVI are pulmonary involvement, skin disease, recurrent systemic inflammation, and developmental retardation. Other clinical features may overlap with monogenic interferonopathies to some degree [7]. Pulmonary involvement was noted in most studied patients (67–82%), which was the main reason for fatalities in SAVI [8, 9]. In our report, the proband and her father were found to have imaging changes in interstitial pneumonia. However, not all patients are symptomatic, as demonstrated by our case, wherein the proband experienced severe respiratory symptoms beginning at a young age while her father was not overtly symptomatic until an adult age. ILD was the prominent manifestation of pulmonary involvement. It occurred early in the disease and was initially insidious, characterized by early onset progressive dyspnea, tachypnea, and/or cough, resulting in pulmonary fibrosis and end-stage respiratory failure. Diffuse alveolar hemorrhage was also reported in the literature [8, 10], which was a main feature of COPA syndrome, another subtype of type I interferonopathy. Ground-glass opacities were the most typical lesions of chest CT, followed by crazy paving patterns, reticular opacities, and cysts. The lesions were frequently asymmetrical compared to other ILD lesions associated with CTD [6, 7, 11]. Pulmonary function tests usually show nonspecific effects, mainly exhibiting a restrictive pattern with diffusion impairment, but may also associate with an obstruction or a mixed lung function impairment [7, 9]. Pulmonary function tests were not performed on this patient due to her young age.

Skin disease of variable severity is displayed in 68.6–86% of patients [2, 7]. Severe skin vasculopathy can present in the nose, cheeks, ears, and acral zones, with extreme skin phenotypes involving perforation of the nasal septum, gangrene of the extremities, and extensive tissue loss. Mild lesions comprise acral and malar rash, purpuric rashes, chilblain lesions, telangiectasia, nail dystrophy, and livedo reticularis. Symptoms of alopecia, sparse and thin hair, and oral mucosal lesions were reported in some cases [7, 11]. Skin biopsies often reveal vasculopathy including perivascular inflammation and vasculitis [1, 12]. SAVI itself may carry an infectious susceptibility [12]. Failure to thrive is a common feature, as weight and height remained below standard deviations [7]. In our case, growth retardation was characterized by substandard height and weight, and skeletal dysplasia, such as delayed fontanel closure and pigeon breast deformity. Besides, articular manifestations are frequent in patients with positive RF. Bone destruction will develop as the disease progresses. In addition, other accompanying symptoms including recurrent febrile attacks, myositis, osteoarthropathy, neurological manifestations, hepatitis, cardiac disease (subepicardial ischemia, pericarditis), and kidney involvement (microscopic hematuria and mild proteinuria) were reported in sporadic cases [7–9, 11, 12].

Laboratory tests of previous studies revealed that SAVI is characterized by systemic inflammation with increased levels of CRP and ESR, which may hardly return to normal [13]. Immune function tests demonstrated hyperimmunoglobulinemia, mainly hyper-IgG and IgA levels despite variable IgM levels. Complement levels were normal in most patients. Mild decreases in CD3 + and CD4 + T lymphocytes and elevations in CD19 + B lymphocytes were frequently observed, while decreased percentages of central memory CD8 + and effector memory CD8 + T lymphocytes with increased percentages of naïve CD4 + and CD8 + T lymphocytes were common [7, 8, 11]. Other common laboratory features of SAVI include positive autoantibodies, mostly high titers of ANA and ANCA with low specificity for MPO and PR3, followed by anti-cardiolipin antibody, lupus anticoagulant, anti-double-stranded DNA antibody, and anti-phospholipid antibody [12]. These autoantibodies highlight the early differential diagnosis with systemic lupus erythematosus or ANCA-vasculitis. Increased RF and CCP were documented, which, when complicated with arthralgia symptoms in early onset, may also confound the diagnosis [14]. High levels of IFN score and STAT1 phosphorylation were recorded in the tested patients [11]. The index case presented typical laboratory features and clinical symptoms. During the 14-month follow-up, the respiratory symptoms were improved, and the chest HRCT was not aggravated after the combined treatment of the baricitinib and steroids. However, improvement of laboratory indicators was limited, especially in autoimmune indices.

Since the first description of SAVI in 2014, a total of 19 types of pathogenic variants in STING1 including p.H72N, p.R94H, p.V147M/L, p.F153V/I, p.N154S, p.V155M, p.G158A, p. G166E, p.C206Y/G, p.G207E, p.R281W (homozygous mutation), p.R281Q, p. R284G/S, and p.K338Rfs*9 have been reported, with 2 (p.R94H, p.K338Rfs*9) of them expressed as uncertain significance [6, 15]. These mutations were heterozygous, and most of them were *de novo*. Previous reports suggested that SAVI remains to be reported as an exclusively autosomal-dominant disease [6]. However, recent reports have presented that autosomal-recessive inheritance caused by homozygous missense pathogenic variants (such as p.R281W) was found in STING1 [16]. Thus far, the genotype-phenotype correlations are not definite due to the limited cases. Jinying Li et al. [17] found that the phenotypes of SAVI in their study have considerable variability compared to the same mutation previously reported. Rohit G. Saldanha et al. [18] suggested that the severity and natural courses of the disease may not relate to mutation type. A recent literature review also returned no obvious phenotypic differences among mutations [7]. As for reasoning, Rensheng Wan et al. supposed that other factors (environmental factors, ethnic background, etc.) in addition to inheritance patterns that resulted in various genotypes-phenotypes should be considered [16]. Nevertheless, a few studies suggested that some clinical features of SAVI may be related to corresponding mutations. Several authors confirmed that patients with the inherited p.V155M variant have a less severe disease course than those with *de novo* variants [1, 16]. A systematic review demonstrated that, compared to patients with the p.V155M mutation, patients with p.N154S mutations had earlier disease onsets and more severe skin lesions, with no differences in respiratory symptoms [2]. These phenomena of heterogeneity on phenotypes were also observed in mouse models [19]. Our case demonstrates the phenotypic heterogeneity of the same genotype in pulmonary disease. The two patients have identical genotypes, but show distinct differences in the onset and progression of their pulmonary manifestations. The proband had early-onset and rapidly progressive pulmonary failure in childhood, while her father had late-onset and slowly progressive pulmonary lesions in adolescence. Moreover, the proband’s father did not suffer from any noticeable growth retardation, which differed from the proband’s phenotype. Above all, the relationships between genotype and phenotype remain for further study.

Currently, there is no standard treatment approach for SAVI. The treatment effects of immunosuppressive agents and biological therapies are mostly limited. Systemic corticosteroids are partially effective in SAVI, yet combination therapies with traditional disease-modifying anti-rheumatic drugs and IVIG had no significant additional effect [2, 11]. Besides, biological agents including TNF-α inhibitors, anti-CD20 antibody, anti-IL-6 inhibitor, and anti-BLyS antibody have disappointing efficacy in SAVI [12, 14]. Based on the disease pathogenesis and the understanding that IFN-I signaling upregulation subsequently activates the JAK /signal transducer and activator of transcription (STAT) pathway, JAK inhibitors have been raised as new potential treatment options. The first use of a JAK inhibitor (ruxolitinib, a selective JAK1/2 inhibitor) in SAVI was reported in 2016 in three cases, which resulted in marked positive effects on skin symptoms, CRP level, interstitial pneumonia, and pulmonary function [20]. Subsequently, the treatment of SAVI with JAK inhibitors has been brought to the forefront and evaluated in more case reports. An overviewing report found that SAVI patients undergoing ruxolitinib treatment before the occurrence of irreversible damage improves clinical features of the disease, especially lung damage [7, 21]. However, there is no consensus on the efficacy of ruxolitinib. Combined treatment of corticosteroids with ruxolitinib in a case report demonstrated rapid improvements in pulmonary hypertension and general well-being as well as resolution of the IFN gene signature, but such treatment coincided with the progression of nasal septal erosion [18]. Moreover, Yan Wang et al. [11] reported that two patients who underwent ruxolitinib treatment exhibited poor responses including a fatality. They speculated that the moribund patient may have succumbed due to the severe disease status and the late introduction of ruxolitinib. Another case with sustained elevation of circulating inflammatory cytokines may have resulted from insufficient dosage. Another selective JAK1/2 inhibitor, baricitinib, was also assessed in SAVI patients. In four cases, baricitinib treatment improved the skin flare, inhibited the further loss of digits, stabilized the ILD and respiratory function, and reduced the requirement of glucocorticoids, while inflammatory markers did not decrease to normal [3]. A recent case report found that baricitinib treatment may have a mitigating effect on disease phenotype, but it failed to prevent further progression of the disease and improve the patients’ IFN signature [16]. Of note, the half-life of baricitinib, the dosage, and the time of administration should be considered when evaluating treatment effects [22, 23]. Baricitinib was chosen in our case and showed some effect on ILD and inflammatory index. However, compared to JAK1/2 inhibitors, the JAK1/3 inhibitor tofacitinib seems to be less effective. Wendao Li et al. [13] reported two SAVI cases treated with baricitinib and tofacitinib, respectively. They both showed an improvement in the disease scores, but baricitinib treatment resulted in a dramatic improvement in lesions of diffuse cords, patchy consolidation, and ground-glass opacities while tofacitinib treatment did not [13]. Although a marked suppression of the IFN signature and therapeutic effects on skin lesions were observed after treatment with tofacitinib [24], the pulmonary defects remained unchanged [25]. Moreover, Xiaolei Tang et al. described two SAVI patients with p.V155M mutations receiving combined treatment of tofacitinib with unsatisfactory consequences [8]. Poor therapeutic effects were observed in improving rashes and ILD and reducing the dosage of steroids. Another patient with early onset SAVI who received short-term tofacitinib treatment died at the age of five months due to respiratory failure [26]. An in vitro study confirmed that tofacitinib could not inhibit dsDNA-triggered, STING-dependent IRF3 phosphorylation in a SAVI model, which suggested that it may not provide an optimal therapeutic intervention to prevent STING-related disease [27]. These studies provide various responses of SAVI patients to JAK inhibitors. The other hypothesis regarding a linkage between the treatment response and a given mutation type needs to be queried in the future, as more SAVI cases will undoubtedly be reported. In addition, we should pay attention to the higher risk of viral infections under the use of JAK inhibitors, as it might aggravate pulmonary fibrosis [16, 28]. Besides, other therapeutic strategies including monoclonal antibodies to IFN-I receptors and STING inhibitors might provide promising therapeutic perspectives [6].

In summary, we describe a case diagnosed as SAVI with severe ILD, which remains stable under the combination therapy of baricitinib and steroids. It seems that baricitinib expressed a therapeutic effect on SAVI in some ways. However, the lung CT image shows no obvious improvement. In general, based on previous literature, JAK inhibitors failed to control the inflammation of the disease completely, which calls for further exploration of JAK inhibitors or other therapeutic strategies, such as monoclonal antibodies to IFN-I receptors or STING inhibitors, to more optimally treat this inflammatory disease.

### Electronic supplementary material

Below is the link to the electronic supplementary material.

Supplementary Material 1

## Data Availability

Important data are within the paper. The datasets generated during current study are not publicly available for privacy reasons, as well as ethical reasons.

## Abbreviations

ILD: interstitial lung disease
IFN: interferon
JAK: Janus kinase
HRCT: high-resolution computed tomography
ANA: antinuclear antibodies
SRP: signal recognition particles
CTD: connective tissue disease
CRP: C-reactive protein
ESR: erythrocyte sedimentation rate
WBC: white blood cell count
ANCA: anti-neutrophil cytoplasmic antibodies
RF: rheumatoid factor
CCP: cyclic citrullinated peptide
IL: interleukin
IVIG: intravenous immunoglobulin
MP: methylprednisolone
