# Supplementary material for: Case report: JAK1/2 inhibition with baricitinib in the treatment of STING-associated vasculopathy with onset in infancy
Source: Pediatr Rheumatol Online J. 2023 Oct 26;21:131. doi: 10.1186/s12969-023-00916-6 (PMC10601276; doi:10.1186/s12969-023-00916-6)
Supplement: Supplementary file 1 — Supplementary Material 1 [file 12969_2023_916_MOESM1_ESM.docx]

Point-by-point Response to Reviewers

Dear Doctors,

We would like to thank all the reviewers for their valuable time and constructive feedback on our manuscript ‘Case report: JAK1/2 inhibition with baricitinib in the treatment of STING-associated vasculopathy with onset in infancy’. We have provided a point-by-point response to the reviewers’ comments in this letter. Our responses and the revised text are highlighted in red in both the letter and the manuscript.

Sincerely,

Meiping Lu MD.Ph.D.

# **Reviewer #1:**

The authors present the case of an infant with SAVI whose father also developed symptoms as a young adult, and describe the partial response of the disease to baricitiinb. However I do not find any new information on clinical findings or the response to treatment that adds to what has already been reported for this autoinflammatory syndrome.

Thank you for your important question.

Since SAVI cases are still very rare worldwide, and the purpose of case reports is to provide as much clinical reference and experience summary as possible for the diagnosis and treatment of the disease, each case report can contribute to the understanding of the clinical characteristics of this disease. To illustrate the similarities and differences between our case and other reported cases, we searched for and summarized all the SAVI cases that received baricitinib treatment. We found 10 relevant articles, which included 17 patients (Table 1). We listed the changes in clinical and laboratory features before and after treatment for all cases, to help with the evaluation.

1. Previous case reports have found that all children improved clinically after treatment, with 9 cases having stable or improved skin lesions, and 11 cases having improved ILD or lung function, which is consistent with our findings. However, our report also included more detailed pulmonary imaging comparisons before and after treatment.
2. One article reported that baricitinib failed to prevent disease progression, but we did not find any evidence of disease progression in our case.
3. Regarding laboratory indicators, previous reports showed that after treatment, ESR was mildly elevated in one case and normal in another, ANA remained positive in one case and turned negative in another, ANCA turned negative in one case, and CRP decreased to normal in one case. In contrast, the laboratory indicators of our case mainly showed that after treatment, ANA, p-ANCA, RF, CCP were still positive, inflammatory markers such as ESR and INF-γ were still mildly elevated, but CRP decreased to normal.
4. Besides, we also reported that high IgG, IgA, IgM and low CD4+ T lymphocyte count did not improve significantly after treatment, which was not mentioned in the above literature.
5. In addition, we have added the relevant content of the same genotype with different phenotypes in the discussion section, mainly pointing out the differences in the onset and progression of pulmonary lesions and the impact on growth in these two cases on page 5, line 39 to page 6, line 2. This perspective is also an insightful and relevant focus for our case report.

Reviewer #2:

Thank you for submitting this article. It is well written and documents two cases of SAVI well. They cases are relatively typical, as is the response to treatment. It provides a good example of varied inter-generational penetrance.

I have a few minor recommendations:

- suggest adding height and weight centiles to better understand growth failure / trajectory

Thank you for this helpful suggestion. I have added the height and weight z scores (SD value) to my paper (on page 2, line 15 and page 3, lines 31 to 32), which can help to better understand the growth failure and trajectory of the patients.

- encephalatrophy is not a widely used term and the MRI brain findings could be summarised with a bit more detail

Thank you for your suggestion. I have replaced the term ‘encephalatrophy’ with ‘widened cerebral sulci and enlarged extra-axial spaces’ on page 2, line 40.

- the generic term trimethoprim / sulfamethoxazole should be used rather than Bactrim

Thank you for this suggestion. I have changed the term “Bactrim” to the more generic term “trimethoprim/sulfamethoxazole” in my paper (on page 3, lines 14 to 15).

- suggest using mg/kg instead of, or as well as, the absolute dose to understand the relative steroid dosing over time

Thank you for this important suggestion. We used mg/kg to express the initial dose of methylprednisolone. After 6 months of treatment, the patient’s methylprednisolone dose was very low (2mg/d), so we also omitted mg/kg for it. We have added mg/kg/d after the specific dose as you advised (on page 3, line 24). Thank you for your comment.

- this case is a good example of differences in phenotypic expression with the same genotype and this should be mentioned in the discussion

Thank you for this important suggestion. I have added the relevant content of the same genotype with different phenotypes in the discussion section, mainly pointing out the differences in the onset and progression of pulmonary lesions and the impact on growth in these two cases. (on page 5, line 39 to page 6, line 2)

Reviewer #3:

This is an interesting case report with literature review on SAVI.

Even if it is only a case description, I think it could be useful for paediatricians and rheumatologists who have to diagnose and/or treat this disease.

Thank you very much for your kind words and positive feedback on my case report. I hope that my case report and literature review can help raise awareness and knowledge of this condition among paediatricians and rheumatologists. Thank you again for taking the time to read and comment on my work.

Reviewer #4:

The manuscript by Wu and coworkers describes the case of a young girl (and his father) with SAVI with lung involvement treated with baricitinib. Although the case is interesting, no innovative elements regarding the clinical course of the disease or the therapeutic approach are reported. The role of JAKi in the management of autoinflammatory type I interferonopathies is well-known. Also, it has been shown that baricitinib can be associated with the stabilisation of lung disease by improving pulmonary function, even though the pre-existing organ damage is irreparable.

Were the pulmonary function tests performed in the patient's father and compared to the previous ones?

Thank you for your question. The patient’s father only had a pulmonary function test at the initiation of baricitinib treatment, and did not repeat it during the follow-up process. We will advise him to complete the pulmonary function test and compare it with the first result at the next visit.

However, this case report do not describe original facts related to SAVI, such as unreported or unusual side effects or adverse interactions involving drugs or unexpected or unusual clinical presentations of the disease. Moreover, English should be carefully revised. Therefore, in my opinion, this manuscript is not suitable for publication in Pediatric Rheumatology.

Thank you for your important question.

Since SAVI cases are still very rare worldwide, and the purpose of case reports is to provide as much clinical reference and experience summary as possible for the diagnosis and treatment of the disease, each case report can contribute to the understanding of the clinical characteristics of this disease. To illustrate the similarities and differences between our case and other reported cases, we searched for and summarized all the SAVI cases that received baricitinib treatment. We found 10 relevant articles, which included 17 patients (Table 1). We listed the changes in clinical and laboratory features before and after treatment for all cases, to help with the evaluation.

1. Previous case reports have found that all children improved clinically after treatment, with 9 cases having stable or improved skin lesions, and 11 cases having improved ILD or lung function, which is consistent with our findings. However, our report also included more detailed pulmonary imaging comparisons before and after treatment.
2. One article reported that baricitinib failed to prevent disease progression, but we did not find any evidence of disease progression in our case.
3. Regarding laboratory indicators, previous reports showed that after treatment, ESR was mildly elevated in one case and normal in another, ANA remained positive in one case and turned negative in another, ANCA turned negative in one case, and CRP decreased to normal in one case. In contrast, the laboratory indicators of our case mainly showed that after treatment, ANA, p-ANCA, RF, CCP were still positive, inflammatory markers such as ESR and INF-γ were still mildly elevated, but CRP decreased to normal.
4. Besides, we also reported that high IgG, IgA, IgM and low CD4+ T lymphocyte count did not improve significantly after treatment, which was not mentioned in the above literature.
5. In addition, we have added the relevant content of the same genotype with different phenotypes in the discussion section, mainly pointing out the differences in the onset and progression of pulmonary lesions and the impact on growth in these two cases on page 5, line 39 to page 6, line 2. This perspective is also an insightful and relevant focus for our case report.

In conclusion, thank you very much for your comments, and I hope the above answers are helpful and satisfactory for your questions.

Table 1 SAVI cases received baricitinib treatment

| Research | Clinical change | Laboratory change | Treatment |
| --- | --- | --- | --- |
| Our case | 1. ILD get improved 2. HRCT scan was stable through the images of the pulmonary changes 3. A catch-up in height | 1. Still positive for ANA, p-ANCA, RF, and CCP 2. Remain high IgG, IgA, and IgM and low CD4+ T lymphocyte counts 3. CRP decreased to normal 4. Levels of ESR (29 mm/H) and IFN-γ (20.1 pg/ml) were still slightly elevated | baricitinib |
| Chia J, Eroglu FK, Ozen S, et al. Journal of the American Academy of Dermatology. 2016;74:186-9. | 1. skin lesions remain stable 2. lung lesion changes not described | NA | baricitinib |
| Sanchez GAM, Reinhardt A, Ramsey S, et al. J Clin Invest. 2018;128(7):3041-3052. | 1. Four patients with vasculitis flares improved with no further loss of digits 2. Three patients with chronic ILD improved 3. A catch-up in height and weight | NA | baricitinib |
| Keskitalo S, Haapaniemi E, Einarsdottir E, et al. Frontiers in immunology. 2019;10:2770. | 1. beneficiary for a vasculitic ulcer, induced hair regrowth and improved overall well-being in one patient took baricitinib | NA | baricitinib |
| Balci S, Ekinci RMK, de Jesus AA, et al. Clinical immunology. 2020;212:108273. | 1. Respiratory symptoms improved 2. Cutaneous manifestations have completely resolved 3. Frequency of fever attacks decreased 4. The literature did not provide images of the pulmonary changes in the patient | 1. Acute phase reactants (CRP, ESR) decreased | ruxolitinib change to baricitinib |
| Clarke SLN, Robertson L, Rice GI, et al. Pediatric rheumatology online journal. 2020;18:37. | 1. Case1 weaned to low flow oxygen 1 month after baricitinib 2. Case2 remains on 1 L per minute low flow oxygen but reports an improvement in activities of daily living after baricitinib 3. The literature did not provide images of the pulmonary changes in the patient | NA | baricitinib |
| Lin B, Berard R, Al Rasheed A, et al. The Journal of allergy and clinical immunology. 2020;146:1204-8 e6. | 1. patient 4, had ILD, delayed gross motor development, failure to thrive, short stature, clubbing was treated with baricitinib with a favorable response 2. Patient 5, had Polyarthritis, clubbing, pulmonary hypertension and reticulonodular densities throughout was treated with baricitinib with a favorable response, but her oxygen dependence continued. 3. Patient 6, had polyarthritis, clubbing, failure to thrive, hair loss and cystic lesions was treated with baricitinib with a favorable response 4. The literature did not provide images of the pulmonary changes in the patient | 1. The changes were not detailed | baricitinib |
| Lin B, Torreggiani S, Kahle D, et al. Frontiers in immunology. 2021;12:636225. | Total five patients   1. Patient 1, her peripheral vasculopathy remained stable, and on PFTs the forced vital capacity improved from 86% to 95% of the predicted value on baricitinib treatment. 2. Weight had reached the 39th percentile, height the 3rd percentile 3. The literature did not provide images of the pulmonary changes in the patient | 1. She developed mild macrocytic anemia, thrombocytopenia, and neutropenia 2. ESR was still elevated (37 mm/h), ANA remained positive albeit at lower titers | baricitinib |
| Cooray S, Henderson R, Solebo AL, et al. Rheumatology. 2021;60:e351-e3. | Case1   1. Weaning of glucocorticoids. 2. His respiratory status remained stable. 3. left eye with perception of hand movements only.   Case 2   1. relief of cutaneous vasculitis feature 2. remains clinically normal 3. Remains blind in both eyes.   The literature did not provide images of the pulmonary changes in the patient | 1. Case1 had the improvement of systemic inflammation (The changes were not detailed) 2. Case2 get normal acute phase reactants (CRP <5mg/l; ESR 14mmHg). | baricitinib |
| Wan R, Fänder J, Zakaraia I, et al. Front Immunol. 2022;13:1029423. | 1. One patient take steroid and baricitinib treatment had a mitigating effect on the disease phenotype, but failed to halt disease progression | 1. Autoantibodies (ANA, ANCA) was negative 2. The IFN signature was still significantly elevated | baricitinib |
| Ladoux C, Pasquet M, Crow YJ, et al. J Clin Immunol. 2023;43(4):699-702. | 1. Enabling weaning off corticosteroids 2. No respiratory signs and symptoms, HRCT scan was stable and pulmonary function tests were normal 3. Catch-up in height and BMI | 1. The interferon signature normalized | baricitinib |
